# Fry's Cocoas and Chocolates

**Published:** 1894-08-25

**Authors:** 


### THE INSTITUTIONAL WORKSHOP.

### PRACTICAL DEPARTMENTS.

### FRY'S COCOAS AND CHOCOLATES.

Those members of the British Medical Association who visited Messrs. Fry and Sons' manufactory at Bristol must have been struck with the advantages in the way of food which the various preparations used by this firm offer to the poorer population of this and other countries. Quite recently a very old cry, which was unfortunately well founded, has been renewed with regard to the bakeries and bakehouses of London and other large cities. Speaking generally, it is not too strong a statement to declare that the conditions under which bread is prepared in this country are well calculated to disseminate disease rather than to promote health. The chief characteristic of Fry's manufactory is that everywhere cleanliness prevails to an extent which is as remarkable as it is satisfactory. Having made a most careful inspection of Fry's roasting-rooms and various departments at Bristol, we are gratified to be able to declare that without a single exception the whole of the arrangements are admirable. Personally we have heretofore had a distaste for chocolate and cocoa, but the beautiful cleanliness which prevailed excited a desire in our minds to overcome this distaste so that we might benefit by the consumption of a food in the preparation of which there are no elements of danger to the consumer. It has been truly said that the poorer classes in England suffer unduly and unjustly from foreign competition. The success which the manufactures of foreign makers of cocoa and chocolate have met with in this country is honourable to the English firms who supply these articles of food. Their desire has always been to supply a pure article, and they have consequently hesitated to interfere with the natural product by endeavouring to make it more soluble by the addition of alkaline matters.

As the result of our personal inspection we are in a position to declare that every consumer of cocoa who uses any of Messrs. Fry's preparations may rest assured that they are absolutely pure, and that they are perfect, so far as it is possible for the manufacturers to make them so. Indeed, manufacturers like Messrs. Fry and Sons, who have expended large sums of money and taken infinite pains to find recipes for the preparation of cocoa and chocolate in various forms for food purposes, are public benefactors of a high order. Some digestions cannot assimilate pure cocoa, whereas they can readily take cocoa with great advantage to their health, provided it is mixed in due proportion with sugar, arrowroot, and other ingredients, and deprived of the superfluous oil. There is a growing practice amongst leading manufacturers to prepare cocoa in combination with other dietetic substances (such as extract of malt) which is coming more and more into general use.

Messrs. Fry and Sons manufacture a good many varieties of cocoa. Chief amongst them we may mention *Fry's cocoa extract*, which consists of pure cocoa deprived of the superfluous oil. *Fry's pure concentrated soluble cocoa*, prepared by a special scientific process, securing extreme solubility, and developing the finest flavour. This brand is pure, easily digested, and should be selected by those who cannot take the ordinary cocoas, because they are less soluble. *Fry's Caracas cocoa* is remarkable for its fine flavour and aroma, and is manufactured with carefully selected cocoas and other ingredients, the perfect wholesomeness of which are guaranteed in accordance with the Act of Parliament. *Fry's malted cocoa* is a combination of Fry's pure cocoa extract (*i.e.*, cocoa deprived of the superfluous oil), with Allen and Hanbury's extract of malt. The malted cocoa does not thicken in the cup, is readily prepared for use, is very palatable, and its nutritive qualities are of a high order. Other preparations are Fry's malted chocolate paste, Fry's improved soluble chocolate in powder, and Fry's powdered chocolate, remarkable for the ease and rapidity with which it can be made ready for table. We have not space to mention the various appetising forms in which chocolate is prepared, but may commend as specially good Fry's wafer chocolate and biscuits, which are supplied in boxes, the chocolate being separate from the biscuit, and so both are made more palatable and appetising. It will thus be seen that habitual users of cocoa may procure any variety from the long list of articles manufactured by Messrs. Fry and Sons. Those who prefer pure cocoa, or soluble cocoa, or recipes which give relatively small proportions of cocoa combined with other food stuffs, will be well advised to support home industries by selecting the brand they prefer from Messrs. Fry's list. We have reason to think that a prejudice may arise against a particular firm's goods because the consumer has not learnt to discriminate as to the preparation most advisable in their individual case. We hope this article may do something to popularise a most useful and invigorating food, the purity and cleanliness of the manufacture of which is guaranteed to all who purchase it from this great Bristol firm.

Finally, we must say a word in praise of the evident care and great taste which have been displayed in preparing Fry's cocoa and chocolate for the market. The contents of many of the tins and boxes are so tempting as to make it hard, if not impossible, to refrain from devouring them with a vigour which might be more flattering to Messrs. Fry than it would be safe for the consumer.

---

DOCTORS' servants—that is, the so-called "valets de pied" of the principal medical men—in Naples are remunerated in a manner quite unique to the locality. They accompany their masters to the patients' houses, and at each visit they are given two francs. Other than this, these particular servants receive no money, which appears to be their time-honoured wages. There is no paucity of applicants for the post of a doctor's servant, we learn, in Naples, so the arrangement appears to be a satisfactory one from their point of view.

